# Can physiological stress alter population persistence? A model with conservation implications

**DOI:** 10.1093/conphys/cot012

**Published:** 2013-06-12

**Authors:** Nina H. Fefferman, L. Michael Romero

**Affiliations:** 1 Department of Ecology, Evolution, and Natural Resources, Rutgers University, New Brunswick, NJ, USA; 2 Department of Biology, Tufts University, Medford, MA, USA

**Keywords:** Allostasis, conservation endocrinology, conservation physiology, glucocorticoids, reactive scope

## Abstract

Recent research has focused on the role of physiological stress in species conservation and population persistence. However, it is currently unknown how much stress individuals can withstand before negative impacts on population size will be detectable. In order to generate testable predictions to address this lack, we created a set of theoretical models that incorporate current theories of how stress, and specifically allostasis (cumulative increase in the cost of coping with stressors), alters an individual's ability to survive and reproduce. Surprisingly, our models predicted the following three non-intuitive results: first, populations where the average individual was exposed to high levels of stress relied preferentially on the oldest and most physically fit individuals for reproduction and population persistence; second, this reliance on the most physically fit individuals led to the average physical condition being highest in the populations where the average individual experienced the most stress; and third, any transient perturbation in the amount of average stress exposure led to a decrease in population size. The mechanism responsible for this decrease was dependent upon the direction of the perturbation; an increase in average stress exposure directly resulted in fewer reproducing individuals, whereas a decrease in average stress exposure indirectly decreased population size via density-dependent feedback. These results have important conservation implications. They suggest that the average physical condition of individuals in a population may be a poor measure of how much stress the population is experiencing, that any disturbance which affects the oldest and most physically fit individuals could have a disproportionate effect on the population, and that any change in the amount of stress experienced by the average individual is likely to have a short-term detrimental impact on the population size.

## Abstract (precis)

A set of general, abstract models generated hypotheses on how wild animal populations respond to stress. We predicted that stressed populations would rely upon the oldest and fittest individuals to reproduce. Consequently, observations of only physically fit individuals may not be an adequate indicator of population health.

## Introduction

Identifying populations at risk of extinction is a primary, but difficult, goal of conservation. One major problem is the prediction of which populations will suffer a decline in size (e.g. population persistence; Reed *et al.*, 2002). In order to be effective, it would be better if the predictions could occur before the real decline takes place. Analytical and simulation models suggest that the dynamics of populations might provide signals of pre-threshold declines (Oborny *et al.*, 2005; Huang *et al.*, 2012), and one laboratory experiment on *Daphnia* reported characteristic, predictable dynamics prior to population decline (Drake and Griffen, 2010). To our knowledge, within-population indicators of imminent decline have not been reported in natural populations, beyond the simple measure of small population size (e.g. Fagan and Holmes, 2006). One alternative to demographic indices is to monitor the physiological condition (e.g. health as measured in different ways) of individuals within these populations, with the assumption that individual decreases in physiological condition will indicate a population at risk (Wikelski and Cooke, 2006). Animals with decreasing physiological condition are often said to be under stress, or responding hormonally and physiologically to noxious internal or external stimuli (called stressors). Conservation biologists have begun to use increases in stress hormones as an index of populations that are at risk (Wingfield *et al.*, 1997; Cockrem, 2005; Walker *et al.*, 2005a), i.e. exposed to more stressors. For the rest of this paper, we will use the term ‘stress’ to refer to the aggregate physiological impact of the stressors to which an animal is exposed. McEwen and Wingfield (2003) would describe this concept as a population-wide increase in allostatic load, and Romero *et al.* (2009) would describe it as a population-wide decrease in reactive scope.

Much is known about the physiological foundations of stress in vertebrates. One of the hallmarks of the stress response is the release of glucocorticoids, which are steroid hormones released into the blood that co-ordinate a diverse array of stress-induced physiological responses (Romero, 2004). Glucocorticoids have proved to be a useful index of stress for several reasons, as follows: they show a gradation of responses, with more severe and more numerous stressful stimuli eliciting greater glucocorticoid release (Sapolsky *et al.*, 2000); they are relatively easy to measure in free-living animals (Wingfield and Romero, 2001; Millspaugh and Washburn, 2004); and increases in glucocorticoids are often correlated with factors of conservation concern, such as pollution (e.g. Hopkins *et al.*, 1997; Hontela, 1998; Wikelski *et al.*, 2001), ecotourism (e.g. Fowler, 1999; Romero and Wikelski, 2002; Walker *et al.*, 2005b), and habitat disturbance by humans (e.g. Wasser *et al.*, 1997; Creel *et al.*, 2002; Homan *et al.*, 2003). Furthermore, increases in glucocorticoids have been linked to population declines (Boonstra and Singleton, 1993; Romero and Wikelski, 2001). Nevertheless, glucocorticoid increases are necessary for animals to survive short-term stressful events, such as predation attempts (Sapolsky *et al.*, 2000), so a small increase in stress (e.g. an increase in local predator density; Scheuerlein *et al.*, 2001) would elicit a normal and beneficial increase in glucocorticoids. What is not currently known is at what point a beneficial increase in glucocorticoids becomes a detrimental increase that could impact population survival.

In order to generate predictions about when an increase in stress starts to have a negative impact on population size, we created an individual-based simulation model (Grimm and Railsback, 2005). Simulation modelling is of growing importance in understanding complex systems (Di Ventura *et al.*, 2006) and is a critical tool in conservation biology because it can be used to predict future conditions, such as the expected results of management activities or of global climate change, and to generate hypotheses to be tested in subsequent research (e.g. Rehfisch *et al.*, 2004; Beissinger *et al.*, 2006). Similar models have been developed to examine the impact of resource allocation and somatic damage (Yearsley *et al.*, 2005) and the impact of senescence (Mangel, 2008). In this study, we simulated populations that differed in the average amount of stress experienced by individuals in that population (i.e. populations differed in their overall exposure to stressors). Within each population, we also included variation among individuals in the amount of stress experienced. Our model tracked population size and individual conditions over time; condition, reproduction, and survival were driven by individual energy acquisition. Individual energy acquisition, in turn, was driven by energy available in the environment, which was density dependent, and by individual stress levels (stress uses energy). This energetics-based approach to evaluating individual condition and the effects of stressors is consistent with the allostasis model of McEwen and Wingfield (2003) and with the reactive scope model of Romero *et al.* (2009).

Our first goal was to use this population model to test the hypothesis that the average physical condition of individuals (based on stress level) in a population provides an accurate reflection of the health, or condition, of the entire population. Here we consider population ‘health’ to be a function of population size and trajectory (i.e. large and increasing populations are healthier than are small or declining ones). We also documented the average age of breeders, as another indicator of population condition. Our other goals in this paper were to investigate the effects of temporary increases and decreases in average stress levels on a population, and again to evaluate the relationship between physical condition of individuals and population health.

## Materials and methods

**Figure 1:**
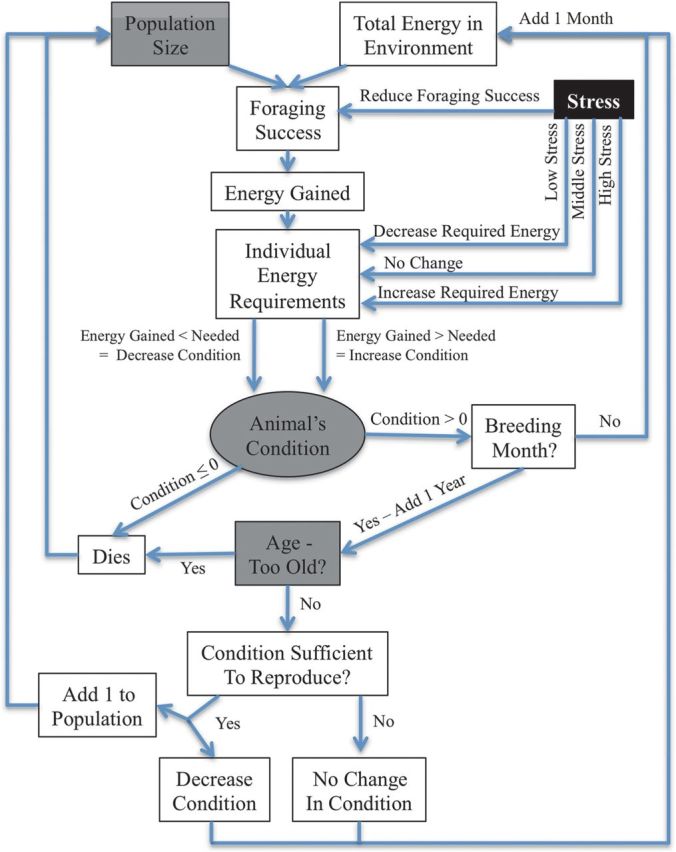
Conceptual representation of the model for each individual at each time step. The logic follows from population-level processes to individual-level processes, and then completes the loop using the state of the individuals as a group as the state of the population. See text for mathematical computations. Blocks in grey indicate variables that were recorded to compare the relative impact of individual traits on the population size. The black block represents the average level of stress in the population and was varied for subsequent runs of the model. The traits in grey were then compared as average levels of stress in the population changed in order to determine the effect of stress on the population.

We implemented an individual-based model (in the C programming language) to capture the population dynamics as individuals in a population forage within their environment, compete with each other, respond physiologically to the impact of stressors by modulating their energetic needs, and survive and reproduce based on maintaining (or failing to maintain) body condition (Fig. 1). We based the variables and parameters of the model on known relationships (Table 1) that we believed to capture the basic processes of competing for and consuming energy from the environment, coping (or failing to cope) with the stressor-induced changes in metabolic needs, and surviving and reproducing over time according to body condition, although the values used were meaningful only in relationship to each other and not as reflections of real measurements. We did this in order to focus on the implications of the most general biological assumptions (rather than attempting to reflect the details of any one particular system), exploring how stress may be expected to affect populations at the most basic levels of biological function. (These basic assumptions, and how they are included in the structure of the model, are summarized in Table 2.)

**Table 1:** Explanation of model components

Parameters and variables	Definition	Examples of sources
*EnvE*(*t*)	Amount of energy in the environment each month	McEwen and Wingfield (2003)
*FRank*_*p*_	Relative foraging success for each individual (*p*)	Smith *et al.* (2001)
*S*_mean_ and *S*_*p*_	Average impact of stress on the population (mean) and individual (*p*)	Sapolsky *et al.* (2000)
*En*_*p*_	Individual energy requirement	Bryant (1997)
*InE*_*p*_(*S*_*p*_) and *DeE*_*p*_(*S*_*p*_)	Increase and decrease in energy in relationship to stress level	Dallman and Bhatnagar (2001) Buttemer *et al.* (1991)
*MaxA*_*p*_	Maximal age for each individual	
*F*_*p*_(*t*)	Energy gained by foraging for each individual each month	
*H*_*p*_(*t*)	Monthly physiological condition of each individual

**Table 2:** All biological assumptions and their mechanism of inclusion in the model. These assumptions provide a very general framework, allowing interpretation of the qualitative behaviour of model results for any system satisfying their description (although for specific quantitative predictions, system-specific parameters and initial variable values would naturally be required)

Assumption	Included in model
There is finite available energy in the environment (fluctuating by time of year)	*EnvE* (*t* mod12)
Individuals are not all equally successful at obtaining energy from the environment	*FRank*_*p*_
Individuals within the population compete with each other to obtain the available energy and, at large enough population sizes, are limited in their success by that competition	*F*_*p*_(*t*)
Individuals have baseline metabolic needs, unaffected by stress	*En*_*p*_
Individuals who experience stress regulate their physiological needs accordingly and, up to a threshold point, this regulation will successfully allow the individual to continue to function uncompromised; beyond this threshold, the individual will be unable to compensate physiologically for the impact of the stressor (this is based on the work of McEwen and Wingfield (2003))	*InE*_*p*_(*S*_*p*_), *DeE*_*p*_(*S*_*p*_), and the threshold points *A* and *B*
An individual's physical condition is dependent on whether or not the energy they obtain from the environment exceeds their energetic needs over time	*C*_*p*_(*t*)
Reproduction involves an energetic cost that exceeds mere survival, and individuals who are failing to meet their own energetic needs will be unable to reproduce successfully	Via algorithmic implementation (description in text of Materials and methods section)
Individuals have a maximal life expectancy	*MaxA*_*p*_

We defined our individual-based discrete, computational model with a monthly time step, *t*. We defined the amount of total energy available in the environment at time *t* according to an annual monthly cycle *EnvE*(*t* mod12), with the energy levels oscillating steadily between a yearly high and low by a constant rate of increase (decrease) from month to month. An individual, *p*, within the total population at time *t* (of size *P*_*t*_) was defined to have a particular constant ranking (*FRank*_*p*_) that described their relative foraging success (individuals are known to have different abilities to gain energy from the environment; see e.g. Gosler, 1996; Smith *et al.*, 2001; Hofer and East, 2003) within the population. This rank was not unique; therefore, individuals were allowed to ‘tie’. Also defined as an inherent property of the environment is the mean impact of stressors to the population as a whole (*S*_mean_; i.e. the impact on the ‘average’ individual in the population). Based on this average impact, each individual *p* was assigned an individual, constant impact (*S*_*p*_) resulting from these stressors (assumed to be normally distributed around *S*_mean_ on a scale from 0 to 50 with a standard deviation of 15, truncated at either extreme).

Based on *EnvE*(*t* mod12), *FRank*_*p*_, and *S*_*p*_, we then defined the amount of energy successfully gained by foraging by individual *p* at time *t* as follows:

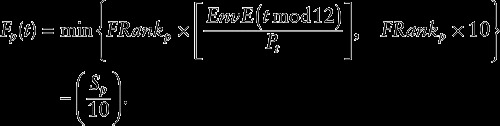

The multiplicative factor of 10 (within the minimum function) was included to cap the impact of the ratio 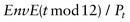 at a factor of 10. This means simply that there is a level of environmental richness beyond which individuals no longer benefit by increasing the energy available per individual (i.e. a resource saturation point per individual). Defined in this way, both density-dependent feedback, as a function of population size, and high average impact from stressors reduced the effective foraging success of individuals in the population (both of which are known to occur; Scheuerlein *et al.*, 2001; Mysterud, 2006).

Individuals were also assigned a constant, baseline physiological energy need (independent of the effects of stressors), *En*_*p*_. This varied by individual (individuals are known to vary in their energy requirements; Bryant, 1997). This baseline, individual physiological need was then acted upon by the influence of stress (as described immediately below), and the resulting total energetic needs of the individual were used to determine the likely survival and reproductive capability of the individual over time as the resources obtained from the environment met, or failed to meet, this need.

We hypothesized that the physiological response to stress functioned in a 2-fold capacity, counterbalancing against itself, first by increasing the energy needs of the individual in order to cope with the stressor (Dallman *et al.*, 1993; Dallman and Bhatnagar, 2001), and second, by decreasing the rate of energy consumption (Buttemer *et al.*, 1991). This, in effect, decreases the energy needs of the individual. We defined the increase in energy need as *InE*_*p*_(*S*_*p*_) and the corresponding decrease as *DeE*_*p*_(*S*_*p*_). We hypothesized that prior to a certain level of stressors (*A*), the ability to slow the rate of energy consumption would be greater than the corresponding increase in energy requirement. Above this level of stressors, the compensatory mechanisms would result in equal differences in the energy needs, thereby effectively cancelling each other's effect. Lastly, there should be a point (*B*) after which *InE*_*p*_(*S*_*p*_) should greatly exceed the counterbalancing effect of *DeE*_*p*_(*S*_*p*_), leading to an energy deficit in the individual. It should be noted that these definitions reflect the empirically motivated understanding that, in response to some stressors, an individual should be able to reduce physiological energy needs. This hypothesis is based on the concept of allostasis proposed by McEwen and Wingfield (2003), which itself is based upon empirical data.

Based on these energy requirements and foraging success, we iteratively defined the physical condition of each individual *p* at time *t* (based on energy alone) as follows: 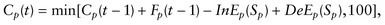, with the initial physical condition of each individual defined at their ‘birth’ as *C*_*p*_(Birth_*p*_) normally distributed around a mean of 55 (out of a possible 100) with a standard deviation of 30. If *C*_*p*_(*t*) ≤ 0, then the individual was said to have ‘died’ and no longer contributed to *P*_*t*_. Otherwise, each individual was allowed to ‘live’ only as long as an individually defined age cut-off at *MaxA*_*p*_ (normally distributed around a mean of 10 years with a standard deviation of 2 years), after which they were also defined to have ‘died’ and were no longer counted towards *P*_*t*_. Each of these values and distributions was chosen to be only sufficiently broad enough to produce a detectable spread in the model outcome; however, any choice of values and distributions held constant across scenarios would result in the same relative success of the populations over time.

While individuals were alive, they were allowed to reproduce once a year (at the seasonal peak in available environmental energy, to reflect empirically observed reproductive patterns in natural populations) providing they had a total physical condition value of at least 30% of the full range and had increased in physical condition during the past month, unless it was already >90% of the full range. Again, these thresholds were held constant regardless of the level of stress experienced by the population, and therefore did not affect model outcome. This requirement for a recent increase in physical condition was incorporated to reflect the hypothesis that even if an individual was generally able to support its energetic needs effectively, a localized inability to do so would reflect a change in the environmental conditions that would decrease the probability of being able to provide for the energetic costs of reproduction successfully. In practice, however, the only cases in which otherwise-successful individuals decreased their condition immediately prior to reproduction were the instances of density-dependent feedback. In these scenarios, all individuals, including those who were utilizing the most energy from the environment most successfully, would experience a decrease in physical condition and be less likely to reproduce.

In order to represent the physiological costs of reproduction, the parent incurred a physical condition cost of 20% of the full range or 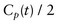 (whichever value was smaller). This modelled the expenditure of energy in order to reproduce (i.e. the increase in allostatic load incurred by an energetically costly life-history event, as defined by McEwen and Wingfield, 2003). At birth, offspring were assigned individual attribute values independent of their parent's values.

Figure 1 presents a flow chart indicating the conceptual construction of the model. In order to compare the relative impact of individual traits on the population size, we recorded not only the population size over time, but also the age and physical condition of individuals able to reproduce. We were then able to compare the average in these metrics with the average amount of stress experienced by individuals in each population.

We followed these simulated populations for enough years to create a stable population structure in an unperturbed environment. In order to discover the effect of an average stress level to the population, the same model was run 51 times, with *S*_mean_ = {0, …, 50}, respectively, exploring all possible levels of stress to the population. We then generated a set of hypothetical intervention (disturbance) scenarios in which the average stress levels of the population were increased or decreased by five points for 1 year and again ran each scenario 51 times, corresponding to each possible average stress level. This five-point change was effected in two different ways. First, an increase in intensity of the current stress burden was modelled by the impact of stress for each individual being temporarily defined to be 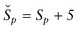. Second, we introduced a novel stressor. Given that different stressors are known to affect individuals in different ways (Sapolsky, 2001), we redistributed the impact of stress *S*_*p*_ for all individuals around a new mean, 
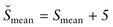
. Due to the stochastic nature of these models, we computed each scenario under 100 Monte Carlo iterations (which we deemed sufficient, because the results from each scenario were seen to converge over replication to within 5% of the population size values). The numbers reported in the figures are representative curves.

When investigating how individual factors (such as age and physiological condition) alter the results, iterative simulation models based on months capture greater specificity of yearly dynamics than do the more common yearly models used in general studies of population persistence (e.g. projection matrix models). The greater specificity was employed in terms of both environmental and individual energetics and was especially useful in modelling the varying temporal scales of survival and reproduction.

## Results

### Basic model

Each model was run while holding environmental energy constant over the 12 months (*EnvE*(*t* mod12) = constant for all values of *t*) as well as while varying environmental energy over a seasonal cycle. The results did not differ from the seasonal model, indicating that local fluctuations in environmental energy available were not an effective limiting factor in the determination of the effects of stress levels on population persistence. Although the average available energy clearly set the thresholds for density-dependent feedback, given that the averages in the seasonal-energy and the constant-energy models were the same, it is not surprising that no difference was observed. We will therefore report only the results from the seasonal model.

**Figure 2:**
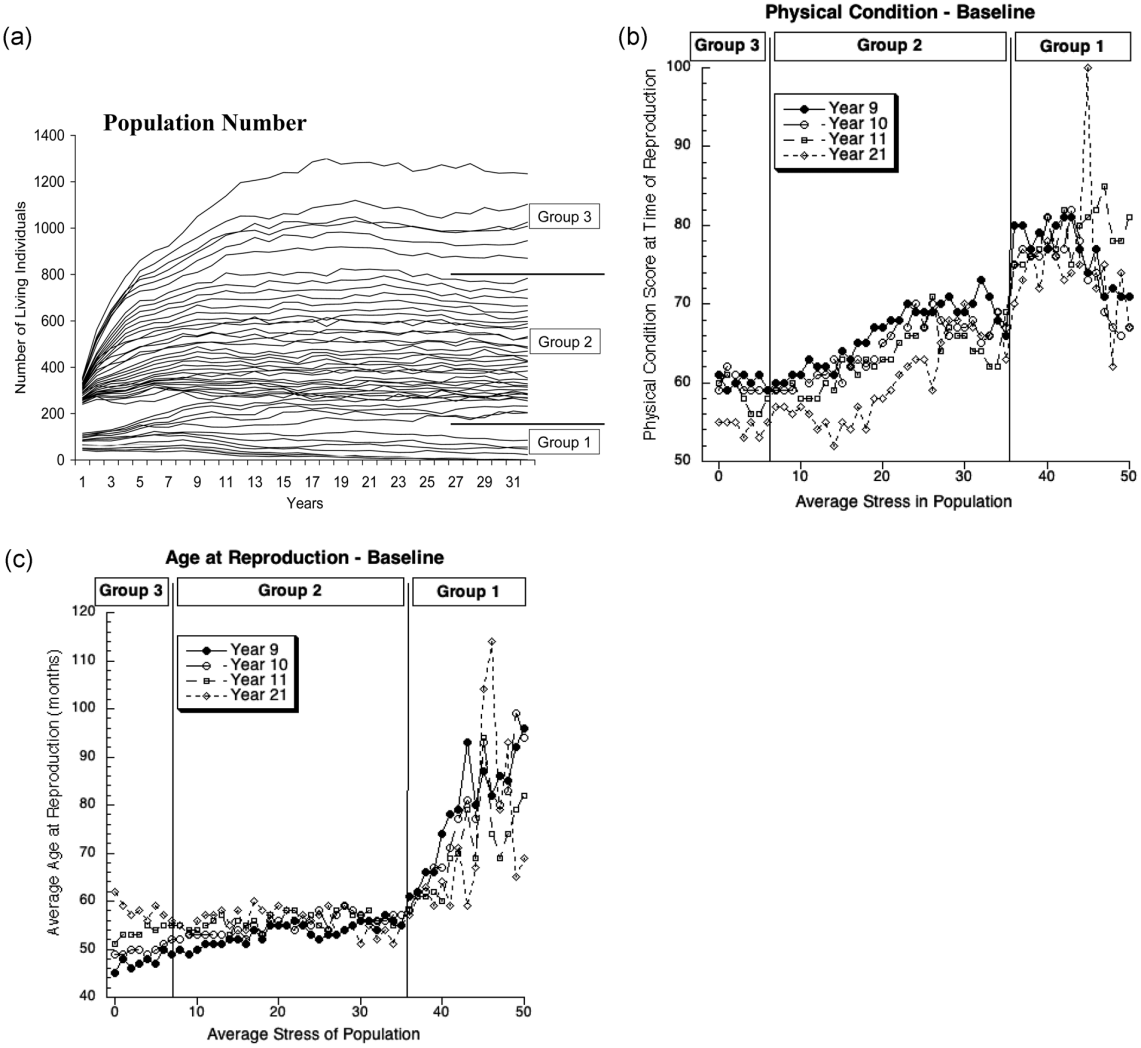
The results for the baseline model scenario starting at the end of the first year. (**a**) The increase in the number of individuals over time for populations, stratified by average stress level from 0 to 50. The Group designations indicate which levels of average stress cause similar patterns of growth. (**b**) The average physical condition of individuals at the time of reproduction experienced in each of the populations (again based upon average stress level) during four different years. Group designations are the same as in (a). (**c**) The average age of individuals at the time of reproduction in each of the populations (again based upon average stress level) during the same 4 years shown in (b). Group designations are the same as in (a). To enable direct comparison, all populations in all models began at the same size.

As predicted, the ultimate population sizes are inversely stratified by the average level of stress affecting individuals in each population (Fig. 2a). Furthermore, we observed three broad groupings of influences of stress on population size. In high-stress conditions (henceforth termed Group 1, encompassing the range of stress levels from 36 to 50), we see very low population sizes (Fig. 2a). Within this group, very small changes in stress level result in proportionately large changes in population size. Past a threshold, there is a second broad group (henceforth termed Group 2, encompassing the range of stress levels of 7–35). Group 2 is still stratified by stress levels, but there are proportionately small consistent changes in population size as stress increases. A last threshold separates Group 2 (with concomitant intermediate stress levels) from the largest populations (henceforth termed Group 3, encompassing the range of stress levels of 0–6). In Group 3, low stress allows large populations, and small changes in stress allow proportionately large changes in population sizes (Fig. 2a).

**Figure 3:**
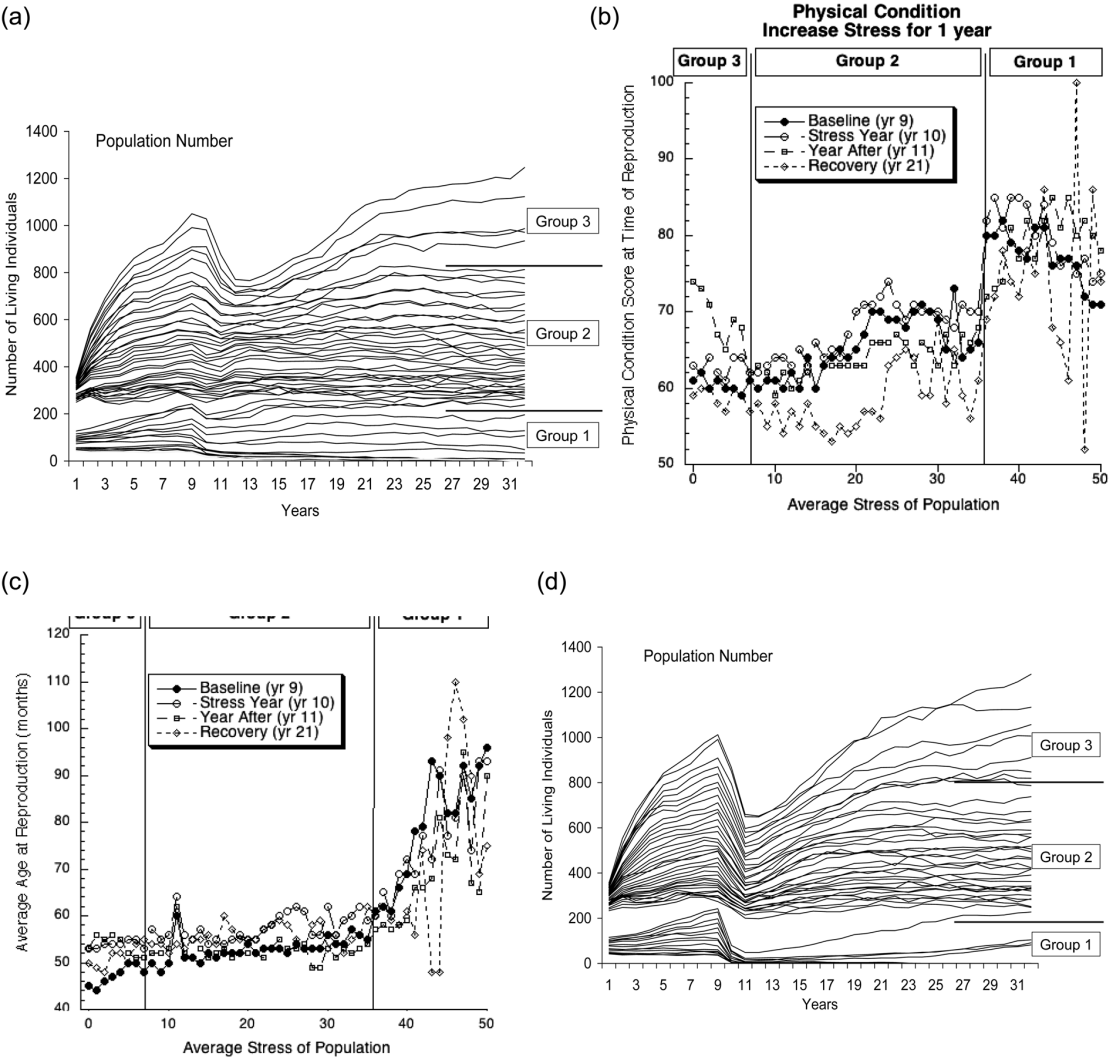
The results from scenarios incorporating a temporary (1 year) increase in the stress experienced by each population. (**a**) The impacts on the size of the populations by the increase in stress. Group designations are the same as in Fig. 2a. (**b** and **c**) The impact on physical condition and age due to the increase in stress during the following 4 years: the baseline (prior to the increase in stress) for each population; the year during which stress was increased; the year directly following the increase (in which the stress levels from before the increase were restored); and the year 10 years after the restoration of the original stress levels. In both (b) and (c), group designations are the same as in Fig. 2a. (**d**) The impact on population sizes caused by the temporary increase in stress combined with the re-assortment of individual stress levels. Again, group designations are the same as in Fig. 2a. To enable direct comparison, all populations in all models began at the same size.

**Figure 4:**
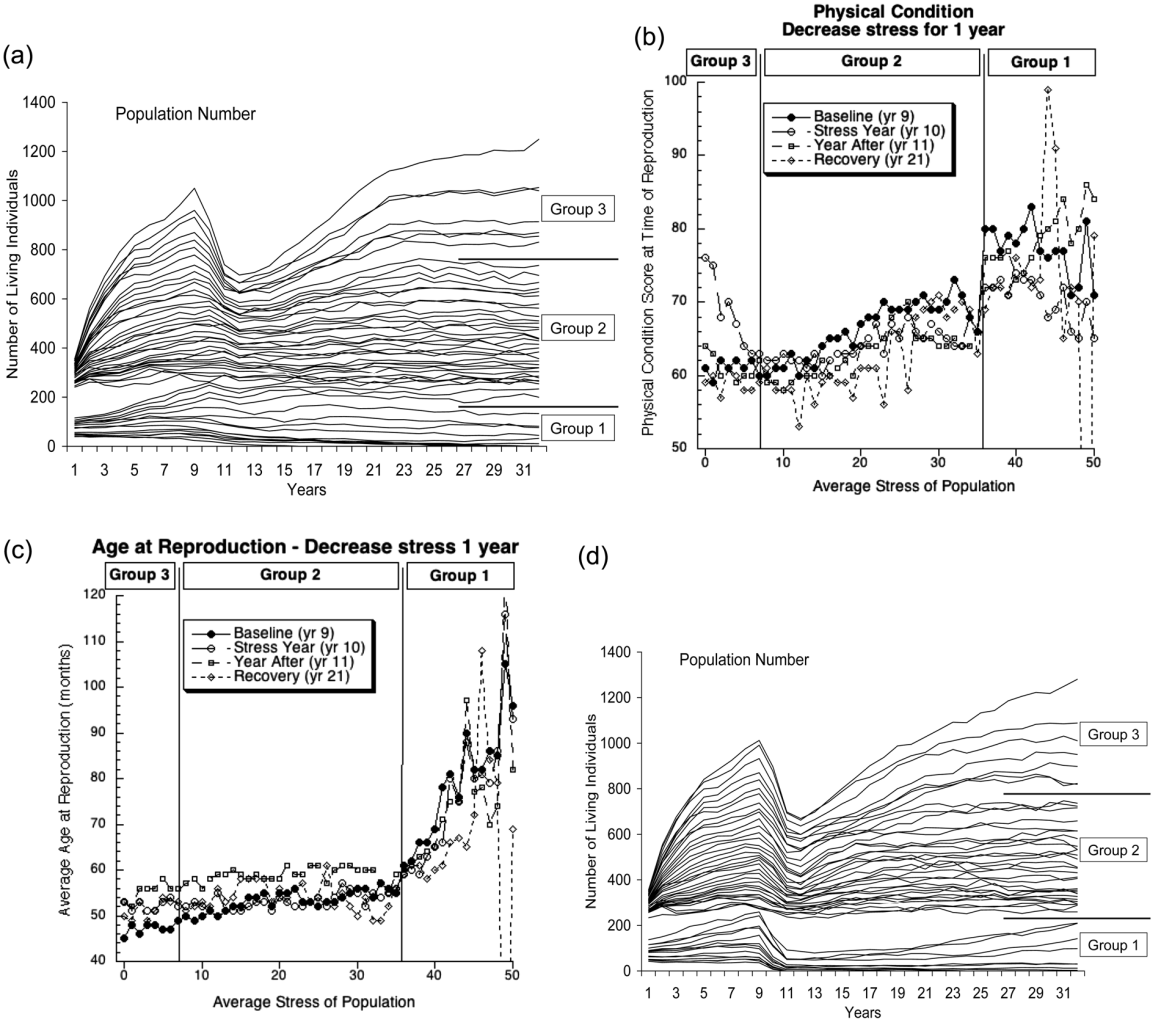
The results from scenarios incorporating a temporary (1 year) decrease in the stress experienced by each population. (**a**) The impacts on the size of the populations by the decrease in stress. Group designations are the same as in Fig. 2a. (**b** and **c**) The impact on physical condition and age due to the decrease in stress during the following 4 years: the baseline (prior to the decrease in stress) for each population; the year during which stress was decreased; the year directly following the decrease (in which the stress levels from before the decrease were restored); and the year 10 years after the restoration of the original stress levels. In (b) and (c), group designations are the same as in Fig. 2a. (**d**) The impact on population sizes caused by the temporary decrease in stress combined with the re-assortment of individual stress levels. Again, group designations are the same as in Fig. 2a. To enable direct comparison, all populations in all models began at the same size.

In conjunction with the overall population size, the average physical condition of the individuals who reproduce in the population increases with the increase in average stress (Fig. 2b). For ease of comparison, and for comparison with Figs 3 and 4, we present results only from years that most accurately examine the intervention studies. Even though population sizes are lower, the physical condition of the reproductive individuals is higher. This results from individuals in lower physical condition being unable to reproduce at the higher stress levels. Likewise, age at reproduction also increases with increasing average stress level (Fig. 2c). However, the increase is much less steep than physical condition scores until a threshold is reached at average stress levels of about 35, after which the age at reproduction increases dramatically. This results from younger individuals dying before being able to reproduce.

### Transitory increase in stress

Figure 2 serves as the baseline for comparing population dynamics when we manipulate the average stress level of each population. When we incorporated a transitory (1 year) increase in stress in each individual (modelling an increase in the intensity of an already-existing stressor after allowing the populations to approach stability at year 10), Groups 1–3 continued to show different behaviours (Fig. 3a). For Group 1, there was an observable decrease in population size, but the overall effect was small and recovered quickly. In Group 2, there was essentially no response, except perhaps at the lowest stress levels. Group 3, however, had a marked response, with population levels dropping by 20–30%. Furthermore, it took ∼10 years for population levels to match control levels, even though the increase in stress lasted only 1 year.

When comparing average physical condition score when reproducing (Fig. 3b), Groups 1 and 2 mimicked the overall population trends; there were no discernable differences in response to the increase in stress. The drop in average physical condition score 10 years after the manipulation (indicated as recovery, year 21) was similar to those seen in controls (Fig. 2) and is likely to reflect the populations reaching density-dependent feedback. The average physical condition scores did not change in Group 3 during the year of increased stress (comparing year 9 with year 10). This was expected because in the model reproduction occurred only once a year when the monthly energy available was at its maximum. However, in the year immediately following the intervention (year 11) the physical condition score increased, indicating that only the more physically fit individuals reproduced that year. By 10 years later, the average physical condition score recovered.

The effects of a 1 year transitory increase in stress on average age of reproduction (Fig. 3c) were similar to the effects on physical condition scores. There were no discernable changes in Groups 1 and 2, and the changes in Group 3 were similar to the change in physical condition scores.

There was a striking difference in population responses when we re-assorted stress levels about a higher mean (Fig. 3d) instead of simply increasing every individual's stress level (Fig. 3a), thereby modelling the introduction of a novel stressor. All three groups then showed a marked decrease in population sizes, with several populations in Group 1 going extinct. Recovery from this 1 year perturbation took ∼10 years.

### Transitory decrease in stress

When we applied a transitory decrease in stress for all individuals for 1 year, there was again no effect in Groups 1 and 2 (Fig. 4a). For Group 3 (and the lower stress level populations of Group 2), there was the same dramatic decrease in population sizes as seen with a transitory increase in stress. In effect, the relaxing of stress allowed many individuals to reproduce, and this immediately brought the population into density-dependent feedback. This became especially acute the following year, when the stress levels increased to their previous level. Once again, it took ∼10 years for these populations to recover.

The effects on average physical condition score (Fig. 4b) and age at reproduction (Fig. 4c) were similar to the effects with a transitory increase in stress. There were no effects in Groups 1 and 2 for either metric, and only the predicted increase in physical condition in Group 3.

When we re-assorted stress scores (Fig. 4d), we again saw population declines in all three groups similar to those caused by a transitory increase in stress (Fig. 3d), but with two important differences. First, it took ∼15 rather than 10 years to recover. Second, in Group 1 the transitory decrease in stress drove fewer populations to extinction.

## Discussion

The major result from these models is that increasing the average amount of stress impacting individuals in a population created a stratified pattern of population sizes. All of these populations could have converged on a single population size (presumably near carrying capacity) had it been the case that each population was able to cope sufficiently with the level of stress present, but they did not. The populations with the highest average stress could not increase their sizes past a certain level. We have chosen in these models to examine the effects of stress on population size. While it is true that these effects do not translate directly into probabilities of population persistence (the main concerns of conservation biology), many sophisticated tools of population viability analysis exist to examine the risks of falling to/below particular threshold population sizes. These tools use the life-history traits of the particular species in question to determine where these risk thresholds may fall for each population. Therefore, in this initial study, we have chosen to focus on the general metric of population size, leaving population viability analyses to examine the implications for the management of particular species.

Furthermore, it was unexpected that stratification due to increasing the average amount of stress would result in three distinct groups. There are reasons in the model for why there might be two thresholds. The first threshold could result from the point at which the ability to compensate for the increased energy needs from stress (Buttemer *et al.*, 1991) outpaces the need (Dallman *et al.*, 1993; Dallman and Bhatnagar, 2001) for the extra energy (point *A* as defined in the model, created to reflect a hypothesized real, individually determined, endocrinological response threshold). In other words, the animal can compensate for stress better than is required, which corresponds to periods of beneficial initiation of stress responses. As defined in the model, this threshold should occur at a stress level of 15. The second threshold could result from the point at which the individual can no longer compensate fully and cannot sustain energy consumption [point *B* as defined in the model, again created to reflect an individual endocrinological threshold that was intended to correspond to allostatic overload in the model of McEwen and Wingfield (2003) or homeostatic overload in the reactive scope model of Romero *et al.* (2009)]. This threshold should be at a level of 35. Note that our transition from Groups 1 and 2 (stress level 36) approximately matches the second threshold, but that the transition from Groups 2 and 3 (stress level of 7) does not match. Even the threshold between Groups 1 and 2, however, is unlikely to be a result only of the model construction. The initial break reflects the point where an individual cannot fully compensate for the energy requirements, but this threshold does not remain distinct. Group 1 continues to stratify and starts to converge into Group 2. Furthermore, when the stress levels after a perturbation were re-assorted (Figs 3d and 4d), the threshold remains, and Groups 1 and 2 do not mix. These two results suggest that the thresholds between groups are emerging properties and not artifacts of the model.

Interestingly, the model seems to explain some of the observations on snowshoe hares (*Lepus americanus*) reported by Boonstra and colleagues. Snowshoe hares undergo a population cycle driven by increases in predation pressure. Increasing predation pressure elevates stress hormones in the hares (Boonstra *et al.*, 1998; Sheriff *et al.*, 2011), so that increasing predation pressure is analogous to the increase in average stress in our model. Similar to results from our model, in the hares the increasing predation pressure results in reduced body condition (Boonstra *et al.*, 1998), lower reproductive capacity (Sheriff *et al.*, 2009), and lower energy availability per animal (Krebs *et al.*, 1995) until a threshold is reached and the hare population collapses. These data show that our model supplements, rather than conflicts with, other published literature on chronic stress.

One of the non-intuitive results from these models is the reliance on older individuals in populations with high average levels of stress (Group 1). In these cases, both the average physical condition of successful breeders and the average age of reproduction increase dramatically as the average stress level of the population increases. In effect, these populations are relying disproportionately on the oldest and most physically fit individuals for reproduction and persistence simply because they are the ones that survive. This result is similar to a population viability analysis of desert tortoises (*Gopherus agassizii*) which found that the oldest females were the most important age cohort for population persistence in this species (Doak *et al.*, 1994). One reason that redistributing stress levels during a perturbation resulted in many extinctions in Group 1 was that many of these important individuals could no longer cope. This result has obvious conservation implications. Populations with high average stress should be very sensitive to any perturbations that impact these important breeders. This impact could be anything (such as a novel stressor) that serves to change the ability of individuals to cope (modelled here by redistributing their stress levels) or something that preferentially targets older individuals (such as hunting).

The reason that physical condition scores decline over time is that the populations are all growing to capacity. At capacity, more individuals at lower physical condition scores get a chance to reproduce because the higher ‘quality’ individuals are already reproducing. This effectively drags down the average physical condition score of the population. Furthermore, in parallel with a reliance on older individuals as discussed above, we can infer from Group 3 that when we increase the average stress in a population, the population shifts to relying more on their most physically fit individuals (Fig. 3b). It would be reasonable to expect a similar shift in Groups 1 and 2. We did not see such a shift in these populations, but the stochastic nature of the model is likely to have masked any effects, because the shifts in Groups 1 and 2 would be predicted to be smaller in these populations with greater average stress. In other words, Group 1, and to a much lesser extent Group 2, are already relying on the oldest and most physically fit individuals, so that small changes affect relatively fewer individuals. In support of this suggestion, note the much higher variation in physical condition and age at reproduction in Group 1.

Individuals die when their stress levels cause long-term energy needs to exceed energy intake, as predicted from the allostasis model of stress (McEwen and Wingfield, 2003). Consequently, our populations grew because only those individuals that were capable of coping with their stress levels survived and subsequently reproduced. However, when we redistributed the stress levels across the population (modelling differential individual responses to novel stressors), in effect we also redistributed the ability to cope. Many previously successful individuals could now no longer successfully cope at their new stress level, and as a result died or failed to reproduce. Given the over-representation of the most physically fit and oldest individuals (discussed above), the redistribution of the ability to cope led to a disproportionately negative impact on these important individuals and led to the dramatic decreases in population size. This provides a mathematical validation that the decreases in population size result from the redistribution of stress to individuals that can no longer cope with their new stress levels. The implications of these data are that populations may be reasonably resistant to increases in the intensity of stressors to which individuals are already accustomed. However, populations may be highly vulnerable to entirely new stressors, even if individuals have been coping reasonably well with high levels of overall stress. This generates predictions such as that populations where individuals are coping reasonably well with habitat fragmentation may not see a decline with further habitat fragmentation, yet may be unable to withstand a new stressor, such as pollution or introduction of a novel predator.

Another non-intuitive result from these models is that, even when stress levels were not redistributed, a decrease in average stress resulted in dramatic population declines. The relaxing of stress allowed many individuals to reproduce and triggered density-dependent feedback. This explains why Group 3 was predominantly affected, because the low average stress levels of these populations meant that they were better able to approach carrying capacity. The implication is that, for healthy populations where individuals are experiencing little stress, any perturbation in stress can impact population size, albeit from different mechanisms. Note also that the population decreases do not threaten these populations with extinction, even though it often takes many years to recover.

### Conclusion

The growing interest in understanding stress physiology in conservation contexts (Cockrem, 2005; Wikelski and Cooke, 2006) is a consequence of the belief that individuals in most, if not all, endangered or threatened populations suffer from increased stress (Wingfield *et al.*, 1997). If individuals in the population are under stress, the results from our model have generated several testable predictions.

First, populations where individuals experience high stress will rely upon the oldest and most physically fit individuals. If true, anything that impacts these individuals will have a disproportionate effect on population size, and the skew will be greatest in the populations with the highest average stress. Empirical data supporting this prediction are currently inconclusive, but there are a few studies suggesting that this might be occurring. An El Nino-induced famine preferentially kills the largest (and oldest) Galapagos marine iguanas (Wikelski and Trillmich, 1997), which are the same individuals that have the highest corticosterone concentrations (Romero and Wikelski, 2001). Although the marine iguana population is considered to be healthy, and El Nino-induced die-offs are considered to be normal, these die-offs do lead to population declines in the immediate subsequent year (Laurie, 1989).

Second, decreases in stress will result in population declines just as do increases in stress, albeit by different mechanisms. To our knowledge, this effect has not been observed directly, but recent empirical data in tuatara (*Sphenodon guntheri*) indicate that relaxing stress (by decreasing specimen collecting) can lead to density-dependent reductions in physiological condition (Hoare *et al.*, 2006).

It is important to remember that this is a general stochastic simulation model tailored to include biologically important parameters derived from the literature on stress. It is not specific to any one species. Rather than beginning with population-specific investigations, based on gathered data and empirical evidence to parameterize our model and validate quantitative predictions for the impact of stressors in one case, we have taken a more general approach. We have focused instead on the types of qualitative behaviours of the most general biological system possible in order to gain insight into the possible impacts, and then be able to be applied (when appropriately parameterized) and tested in any population of interest. Many efforts in biology have been of this form, producing valuable theoretical insights from studying the qualitative difference in system behaviours under different general assumptions [cf. Hamilton's rule (Hamilton, 1964a, b) and the epidemiological models of Kermack and McKendrick (1933)], and there have been recent calls for more such simulation models (e.g. Di Ventura *et al.*, 2006). We have attempted to follow in their footsteps by using very general (and relationship-driven rather than data-driven) models to provide a framework by which the impact of stressors on populations may be considered, and to use this framework to generate empirically testable hypotheses for many systems of interest. Hopefully, the results of these initial models will lead to further work testing the derived predictions and refining the models.
